# Ion-Exchange Membranes and Processes

**DOI:** 10.3390/membranes11110814

**Published:** 2021-10-26

**Authors:** Natalia Pismenskaya, Victor Nikonenko

**Affiliations:** Membrane Institute, Kuban State University, 350040 Krasnodar, Russia; v_nikonenko@mail.ru

Synthetic ion exchange membranes (IEMs) are made from organic, inorganic, or mixed and composite materials. IEMs have not yet reached the success of biological membranes, but they largely reproduce their functions or even possess such unique properties that biological membranes are not endowed with. There is a significant variety of IEM types. Cation-exchange monopolar membranes selectively transfer cations, and anion-exchange monopolar membranes selectively transfer anions. Mosaic IEMs contain alterna- ting cation and anion exchange zones, and amphoteric IEMs are a mixture of cation and anion exchange materials. Modification of the surface of monopolar IEMs with highly crosslinked ion exchange materials or materials that contain polar groups, the electric charge of which is opposite to that of the fixed functional groups of the substrate membrane, provides specific selectivity for certain ions. Bipolar membranes, which contain cation-exchange and anion-exchange layers, are capable of generating protons and hydroxyl ions from water at the interface of these layers. A similar phenomenon (water splitting) can be enhanced or suppressed at the monopolar IEM/solution interface by varying the catalytic activity of fixed functional groups with respect to the water dissociation reaction. The imparting of hydrophobic properties to the surface of IEMs and the purposeful design of geometric and electrical inhomogeneity stimulates the development of electroconvective vortices in the solution adjacent to the membrane in an electric field. Some of the IEMs are both electronically and ionically conductive. Additionally, this is not a complete list of membrane types and capabilities. The various properties of IEMs make them very attractive for the development of low reagent and environmentally friendly techno-logies for the purification, separation and concentration of various substances, as well as the reagent-free conversion of acids and alkalis into salts and back again, for use in low-temperature fuel cells, potentiometric sensors, microfluidic devices, etc.

The aim of the Special Issue “Ion-Exchange Membranes and Processes” was to obtain a holistic picture of the latest advances in the synthesis and properties of new ion-exchange materials, the methods of modification of commercial and experimental IEMs, the experimental and theoretical study of the phenomena, which accompany the ion transfer in membrane systems under the action of an electric field and in its absence, as well as the use of these membranes in various processes.

The articles published in this Special Issue reflect the main trends in the development of fundamental and applied aspects of membrane science. 

The relationships between the manufacturing method, material composition and membrane structure are studied by Akberova et al. [1]. In the example of heterogeneous IEM Ralex CM Pes manufactured by MEGA a.s. (Czech Republic), it has been shown that an increase in the milling time of resin grains from 5 to 80 min leads to a more than 1.5-fold decrease in the resin grain radius and in the distance between them, as well as a decrease in the size of pores and structure defects between grains and inert binders of polyethylene. The two-fold growth of the conducting fraction on the surface and appro-ximately a 40% increment in this fraction in the cross-section are observed if the resin loading increases from 45 to 70 wt % in the resin/polyethylene ratio. However, the growth of the conducting fraction is accompanied by an increase in the macropore fraction and structure defects on the membrane surface. These results can help manufacturers of he-terogeneous membranes to select the optimal ion exchange resin loading and grinding time. In addition, these data provide a key for designing the surface of IEMs with a given morphology (dimensions of conductive and non-conductive zones), which are important for the development of electroconvection, as well as for increasing the selectivity of heterogeneous membranes due to the reduction in macropores and defects in the structure of their surface and volume.

In Ref. [2], Parshina et al. continue a series of works aimed at developing Donnan potential (DP) sensors (analytical signal is the Donnan potential). They investigated, in a wide pH range, the influence of the incorporation of dopants with proton-acceptor pro-perties (a silica, surface modified by 3-aminopropyl and 3-(2-imidazolin-1-yl)-propyl groups) into perfluorosulfonic acid cation exchange membranes (MF-4SCandNafion), and their treatment conditions on the characteristics of DP sensors in aqueous solutions containing asparaginate and potassium ions. The relationship between the water uptake and diffusion permeability of pristine and modified IEMs, conditions of their treatment and the cross sensitivity of developed DP sensors to counter and co-ions were studied. The results of these studies have demonstrated that the use of these sensors ensures the accuracy of asparaginate determination in the drug commensurate with the accuracy of known voltametric sensors. The simplicity, high stability, relatively low pharmaceutical dilution, and reagent-free analysis make the developed DP sensors very promising for the analysis of pharmaceuticals.

Wei et al. [3] proved the highly efficient and cost-effective use of conventional electrodialysis for a high degree of purification of methylsulfonylmethane (MSM) from mineral (NaNO_3_) impurities. This conclusion is based on studies of the influence of the voltage drop values, the concentrations of MSM and sodium nitrate, the assessment of energy consumption and the cost of the electrodialysis demineralization process.

Much of the research published in this Special Issue has focused on the transport mechanisms of ampholytes in systems with IEMs and on the application of various membrane methods for processing ampholyte-containing solutions. Ampholytes are a wide range of substances that are involved in the protonation–deprotonation reaction with a solvent (water), as well as with each other, and are capable of changing the electric charge depending on the pH of the medium. This property predetermines the particular attractiveness of the processes with IEMs (in which pH regulation can be carried out without the addition of reagents) for the extraction and purification from mineral impurities of proteins, amino acids, particles of polybasic organic and inorganic weak acids and other ampholytes. At the same time, the ability of ampholytes to change the electric charge depending on the pH of the medium and the simultaneous presence of ampholyte particles, which have different electric charges, in the processed solutions significantly complicates the mechanisms of mass transfer in membrane systems in comparison with the known ones for strong electrolytes.

Pismenskaya et al. [4] obtained the concentration dependencies of diffusion permeability of homogeneous and heterogeneous anion-exchange membranes (AEMs) in solutions of ampholytes (sodium bicarbonate, NaHCO3; monosodium phosphate, NaH2PO4; and potassium hydrogen tartrate, KHT). They showed that the diffusion permeability increases with dilution of the ampholyte solution while, in the case of strong electrolytes (sodium chloride, NaCl), it decreases. The enrichment of the internal AEM solution with multiple charged counterions (1) and the increase in the pore size of AEMs (2) with dilution of the external solution are identified as the main reasons for the unusual concentration dependencies of the diffusion permeability of membranes in the case of ampholytes. Phenomenon (1) is caused by the Donnan exclusion of protons, which are the products of protolysis reactions. Phenomenon (2) is caused by the stretching of the elastic polymer matrix due to the penetration of strongly hydrated anions of carbonic, phosphoric, and tartaric acids into the AEMs. These results shed light on the reasons for the faster destruction of membranes used in dialysis and electrodialysis processes of wine conditioning, whey demineralization, phosphate recovery from wastewater, etc., compared to the case of strong electrolytes.

A simple non-steady state mathematical model [5] takes into consideration the ability of the amino acid to enter the protonation/deprotonation reactions. This model is proposed for the process of purification of an amino acid solution from mineral salts by batch recirculation neutralization dialysis (ND). The model takes into account thickness, exchange capacity and electric conductivity of IEMs and concentration, component nature and flow rate of the solution in dialyzer compartments. In contrast to the known models, the new model considers a local change in the ion concentration in membranes and the adjacent diffusion layers. The validity of the model was confirmed experimentally using a laboratory dialysis cell formed by profiled heterogeneous cation and anion exchange membranes. The non-steady-state ND process is carried out using a strong electrolyte (NaCl) solution or equimolar mixture of NaCl and ampholyte (phenylalanine) solutions. The model adequately describes the ND of a strong electrolyte (NaCl) and amino acid (phenylalanine) mixture solutions in the case where the diffusion ability of amino acids in IEMs is much less than mineral salts.

Kadel et al. [6] analyzed the capacity of six polyether sulfone filtration membranes (FMs) with molecular weight cut-offs (MWCO) of 5, 10, 20, 50, 100 and 300 kDa to separate peptides from a complex whey protein hydrolysate using electrodialysis with filtration membranes (EDFM). They show, interestingly, that global peptide migration to both recovery compartments increased linearly as a function of MWCO. However, peptide selectivity changed according to the recovery compartments and/or the peptide’s charge and molecular weight with an increase in the MWCO of FMs. This was the first time that the significant impact of charge, MWCO and pore size distribution of polyether sulfone filtration membranes on a wide range of MWCO was demonstrated on EDFM performances. These results will help broaden the scope of a green and promising technology for bioactive peptide fractionation.

Dufton et al. [7] focus on suppression scaling and fouling by the application of a pulsed electric field (PEF) in electrodialysis to produce dryable acid whey. For the first time, PEF parameters of relatively low frequencies (<1 Hz) were studied in underlimiting current modes for electrodialysis of a complex solution such as acid whey. The optimal duty cycle and current pulse amplitudes were selected among the eight investigated modes. It was found that longer pauses between current pulses lead to the better demi-neralization of divalent ions. The main mechanisms of the PEF effect on concentration polarization, water splitting and scaling are discussed.

Grushevenko et al. [8] studied the effect of CO_2_ loading of the lean 30 wt % mono-ethanolamine (MEA) solution on the efficiency of heat stable salt (HSSs) removal using a two-stage electrodialysis unit. These HSSs were formed and continuously accumulated in the amine-based solvents due to solvent degradation and impurities (SOx, NOx, etc.) in the feed gas. It was shown that the transport of HSS anions in AEMs decrease in the following order: nitrate > formate, acetate > sulfate, oxalate, which is governed by the anion charge and the protonation–deprotonation reaction constants. A relationship was established between the content of carbon dioxide in the feed solution and energy consumption for electrodialysis removal of HSSs.

## Data Availability

Not applicable.
